# Is Left-Handedness Associated With Greater Success From the 7-Meter Line? An Analysis of 7-Meter Records Across Various Handball Competitions

**DOI:** 10.1177/00315125241272503

**Published:** 2024-08-12

**Authors:** Aron Laxdal, Tommy Haugen, Ørjan Angeltveit, Christian Sørensen, Andreas Ivarsson

**Affiliations:** 1 Department of Sport Science and Physical Education, 4343 University of Agder, Kristiansand, Norway; 2 Center of Research on Welfare, Health and Sport, 3694 Halmstad University, Halmstad, Sweden

**Keywords:** handball, laterality, negative frequency-dependent advantage, penalty throws

## Abstract

While left-handers have been overrepresented among 7-meter shooters in handball, previous investigators have not found success from the 7-meter line to be related to handedness. Drawing on previous handedness research in sport, we performed two studies to examine possible negative frequency-dependent advantages to left-handedness during 7-meter throws among elite players. In Study I, we analyzed the records of 974 7-meter shooters from Danish and Norwegian elite divisions (485 males and 489 females) and found that left-handed males were overrepresented compared to the prevalence of left-handers in these two leagues, but left-handed females were not. An analysis of covariance showed no statistically significant associations between throwing arm or sex, and success from the 7-meter line. In Study II, we analyzed the records of 899 7-meter shooters at 41 major championships for both males and females between 2007-2023 (442 males and 457 females). We again found left-handed males to be overrepresented compared to their prevalence at the championships, but left-handed females were not. Also, in alignment with Study I, an analysis of covariance found no associations between throwing arm or sex, and success from 7-meter throws. These findings further underline the complexities associated with lateral biases in sports, where there appear to be benefits for left-handed males in the selection process that are not evident during performance execution.

## Introduction

Several investigators have found that being left-handed can be advantageous in some sports, resulting in an overrepresentation of left-handers among elite athletes (Chance & Maymin, 2023; Connor et al., 2020; Harris, 2016; Loffing, Schorer, et al., 2012; Loffing & Hagemann, 2015, 2016; Richardson & Gilman, 2019). While different explanations for this lateral bias have been proposed, there has been a growing consensus that lateral advantages result from innate abilities or negative frequency-dependent factors (Groothuis et al., 2013; Loffing & Hagemann, 2016). In other words, left-handers either perform better because they are genetically predisposed to having quicker reactions, being more aggressive, and having better spatial awareness than right-handers, or they have an advantage because their movement patterns are infrequent and less easily recognized by opponents (Loffing & Hagemann, 2016). However, there are indications that both innate abilities and negative frequency-dependent advantages may play a joint role in explaining the advantage (Loffing & Hagemann, 2015; Pollet et al., 2013; Witkowski et al., 2019).

Previous work from various sports has shown that the significance of lateral biases can vary with sport context and such athlete variables as proficiency level, sex, and weight-class, among other factors (Dochtermann et al., 2014; Loffing, 2017; Loffing & Hagemann, 2015, 2016), indicating that the underlying mechanisms for lateral biases are complex and multifaceted. Richardson and Gilman (2019) also postulated that their chosen statistical approach (i.e., analyzing individual records as opposed to individual events) was at least partially responsible for their findings, suggesting that different statistical approaches should be explored before drawing any definitive conclusions about possible relationships between laterality and sporting success.

Handball is an interesting context in which to study laterality, because this sport relies on highly one-sided movement patterns that may offer inherent tactical benefits to left-handers. All teams strive to have two left-handers on the court at any time, since players who throw with their left hand have favorable shooting angles on the right side of the field. These presumed tactical advantages, coupled with the relative paucity of left-handers in society, ensure that left-handers are favored during selection processes (Baker et al., 2013; Karcher & Buchheit, 2017; Papadatou-Pastou et al., 2020).

However, the 7-meter shot is one action during handball games where there are no presumed handedness-related angular advantages, since 7-meter shots are executed from a fixed angle, and as the name indicates, are always seven meters from the goal. This free penalty shot is awarded to the attacking team when a defender robs them illicitly of a clear goal scoring opportunity. No defenders are allowed to interfere with the shot, leaving only the goalkeeper to obstruct it and prevent a goal from being scored. Since 7-meter shots make up a substantial number of shots in any given game (Laxdal, Ivarsson, Sigurgeirsson, & Haugen, 2022), and the likelihood of scoring from a 7-meter shot is quite high (Laxdal et al., 2024), 7-meter shooters should be chosen with care and deliberation.

Even though left-handed handballers are expected to be overrepresented when compared to their frequency in the general population, they appear to take a disproportionately large proportion of 7-meter shots, meaning that coaches are more likely to place left-handers than right-handers at the 7-meter line (Laxdal et al., 2022b, 2023; Lobinger et al., 2014). However, when looking past their overrepresentation, previous investigators have not found left-handers to be more likely to score from 7-meter shots than right-handers (Laxdal et al., 2022b, 2023), signalling that coaches may be choosing best shooters, regardless of their handedness. In line with Richardson and Gilman’s (2019) arguments, we sought to test whether analyzing a sample of players’ indvidual records instead of a sample of 7-meter events would yield different 7-meter success rates between left-handers and right-handersand shed new light on this relationship. Seeing as any advantages were likely to be small, we utilzed larger datasets than have been tested before to increase statistical power for detecting small effect sizes.

Our overall aim was to examine the 7-meter records of elite 7-meter shooters to examine: (a) whether left-handed shooters were overrepresented in this sub-group compared to their expected representation, (b) the relationship between throwing hand and success at the 7-meter line, and (c) whether there were any sex-differences in left-handed versus right-handed success rates. We used a two-study approach based on two different large data samples. We examined shooters from the Danish and Norwegian elite divisions from 2015-2023 in Study I, and we examined shooters from all European Championships, World Championships and Olympics from 2009-2023 in Study II.

We chose the Danish and Norwegian leagues for Study I for two reasons. First, these leagues are among the very best in the world, currently sitting in the 1st, 5th, 5th, and 9th place on their respective EHF coefficients (European Handball Federation, 2023). Second, the data available on 7-meter shots on two websites, https://www.tophaandbold.dk/ and https://www.handball.no/, represent some of the largest longitudinal public domain collections of 7-meter shooter records at the club level. We chose the European Championship, the World Championship and the Olympics for Study II, as they are the pinnacle championships of national team handball, and the 7-meter records of all shooters from the most recent tournaments are available online (on the official websites of the European Handball Federation and the International Handball Federation, respectively).

## Method

### Ethical Considerations

As both studies were based on publicly available data, no informed participant consent was needed.

### Study I Data

The 7-meter records of each player who took a 7-meter shot in the Danish elite divisions (male and female) since 2015 are listed on the official web page of Danish elite handball (https://www.tophaandbold.dk/) on a season-to-season basis, with eight seasons available when data were collected (2015–2023). We collated records from all eight seasons into a single score for each player. Regarding the Norwegian leagues (male and female), data are available in the play-by-play on the official webpage of the Norwegian Handball Federation (https://www.handball.no/). We used Python (3.9.12) and Jupyter Notebook (6.5.8) to scrape and collate all 7-meter shots by all individual players.

In total, 974 different shooters (509 from the Norwegian leagues, 428 from the Danish leagues; 485 males and 489 females) executed 33,856 7-meter shots in the eight-year period. As would be expected, there were large variations in the number of attempts by individuals, with 200 players taking only a single 7-meter shot and one player taking 535 7-meter shots (*Mdn*_shots attempted_ = 10, IQR = 42).

### Study II Data

We collated the 7-meter shot records of all male and female 7-meter shooters during the 41 major championships between 2007-2023 (i.e., European championship, World Championship and Olympics; the 2023 women’s world championship was not included, as it was not finished when data collection took place) into a single score for each player. Regrettably, only data for the top 40 goal scorers were available for many of the older tournaments (2008-2014 European Championships, 2007-2015 World Championships, and 2008-2012 Olympics). This data set includes 899 different 7-meter shooters (442 males, 457 females) who collectively took 16,398 7-meter shots, with 149 players taking only a single 7-meter shot and one player taking 248 (*Mdn*_shots attempted_ = 8, IQR = 19).

### Variables in Both Studies I and II

In addition to the players’ 7-meter shooting records (calculated by dividing the total number of 7-meter goals by the total number of 7-meter shots), the variables of interest were the players’ dominant throwing arm (categorized dichotomously into left- and right-handed), sex (male or female depending on their competition category), and number of 7-meter shots the shooters performed. Information regarding the players’ dominant throwing arms was gathered using publicly available information (photographs, videos, and player registries) that was independently corroborated (in Study I by four goalkeepers from the respective leagues; and, in Study II, independently by two members of the research team).

### Statistical Analyses

To determine whether left-handed shooters were overrepresented within these samples, we performed binomial tests of proportions using SPSS (version 29.0; IBM Corp., Armonk, NY). In Study I, this comparison was between the number of left-handed 7-meter shooters in the data sample and the average number of left-handed players in the respective leagues, using the first gameday of the 2022/2023 season as a proxy for the other seasons. This average proportion of left-handers in the leagues was 25.1 % for males and 22.7 % for females, both of which exceeded the typical 10% of left-handers in the general population. In Study II, this comparison was between the average number of left-handed players at the major tournaments, with the 2020 Olympics serving as a proxy for all tournaments. The average proportion of left handers at that tournament was 26.1 % for the males (similar to the percentage reported in Laxdal, Ivarsson, Þorgeirsson, & Haugen, 2022) and 22.7 % for the females (similar to our findings in the Study I data set).

To predict shooting success from 7-meter shots, we ran an analysis of covariance (ANCOVA) in SPSS (version 29.0; IBM Corp., Armonk, NY) with three independent variables, where sex and number of 7-meter shots were designated as covariates. As noted, the distribution of shot attempts in both studies was skewed, with more players shooting few 7-meter shots. Accordingly, we performed a sensitivity analysis to account for any success rate differences between those who took many shots (the highest third shot >27 shots in Study I and >16 shots in Study II) and those who took few shots (the lowest third shot <3 shots in both studies), or we took a moderate number of shots (the middle third took 4-26 shots in the Study I data set and 4-16 in the Study II data set). Due to concerns related to the assumptions for ANCOVA, we performed bootstrapping (n iterations = 5000). In all analyses, we set *p* < .05 for statistical significance. Effect sizes were calculated using partial eta squared (η^2^; .0099 = small, .0588 = moderate, .1379 = large; Richardson, 2011).

## Results

### Study I

As expected, most of the 974 shooters from the Study I data set were right-handed (74 %). The throwing arm of six shooters (<1 %) could not be determined, and they were excluded from further analyses, leaving 718 right-handers and 250 left-handers to be analyzed. Binomial tests of proportions of right- and left-handers revealed that left-handers were overrepresented as 7-meter shooters, relative to their prevalence in the male leagues (29.5 % [142 players] vs. 25.1 %, *p* = .017), but left-handers were not overrepresented among female 7-meter shooters, relative to their prevalence in the female leagues (22.2 % [108 players] vs. 22.7 %, *p* = .426).

Most of the 33,856 7-meter shots analyzed were successful (77%), and there were nearly equal percentages of successful shots from right-handed shooters (76%) and left-handed shooters (77%). The results of the ANCOVA on 7-meter shot successes showed no significant main effect for throwing arm (*F* [1, 964] = .12, *p* = .729, η^2^ < .001). Sex was not a statistically significant covariate (*F* [1, 964] = 1.51, *p* = .219, η^2^ = .002), while number of 7-meter shots were (*F* [1, 964] = 37.02, *p <* .001, η^2^ = .037). The sensitivity analysis revealed no statistically significant effects when it came to throwing arm (lowest third: *F* [1, 318] = .82, *p* = .367, η^2^ = .003; middle third: *F* [1, 316] < .01, *p* = .955, η^2^ < .001; highest third: *F* [1, 325] = .15, *p* = .700, η^2^ < .001). While there were statistically significant sex differences when comparing males and females in the highest third (lowest third: *F* [1, 318] = .01, *p* = .918, η^2^ < .001; middle third: *F* [1, 316] = 3.89, *p* = .050, η^2^ = .012; highest third: *F* [1, 325] = 4.93, *p* = .027, η^2^ = .015), the effect size was small.

### Study II

Among the 899 7-meter shooters from study II, 70% threw with their right hand, and the throwing arm of five shooters (<1%) was unknown (leading to the exclusion of their data from further analyses). The binomial test of proportions indicated that left-handed males (*n* = 157) were overrepresented compared to their actual prevalence in the tournaments (35.5 % vs. 26.1 %, *p* < .001), while no left-handedness overrepresentation was found among the females (n left-handed players = 111; 24.6 % vs. 22.9 %, *p* = .216).

Of 16,398 shots, 77% were successful. Again, the percentages of successful shots from right-handed shooters (76 %) and left-handed shooters (78 %) were similar. According to the ANCOVA, there was no significant main effect on 7-meter shot success for throwing arm (*F* [1, 890] .225, *p* = .635, η^2^ < .001). Sex was not a statistically significant covariate (*F* [1, 890] = 3.09, *p* = .079, η^2^ = .003), while number of 7-meter shots was (*F* [1, 890] = 32.67, *p* < .001, η^2^ = .035). There were no significant effects for throwing arm (lowest third: *F* [1, 304] = .69, *p* = .41, η^2^ = .002; middle third: *F* [1, 286] = .04, *p* = .850, η^2^ < .001; highest third: *F* [1, 295] = .48, *p* = .487, η^2^ = .002) or sex (lowest third: *F* [1, 304] = 3.24, *p* = .073, η^2^ = .011; middle third: *F* [1, 286] = .19, *p* = .660, η^2^ = .001; highest third: *F* [1, 295] = .16, *p* = .693, η^2^ = .001) in the three separate groups of 7-meter shooters, based on their frequencies of throwing 7-meter shots.

## Discussion

In this study we explored whether being left-handed was advantageous for handballers taking 7-meter shots. In line with Laxdal, Ivarsson, Þorgeirsson, and Haugen (2022) and Lobinger et al. (2014), we found left-handed males to be overrepresented among 7-meter shooters at the world class level. This stands in contrast to Laxdal et al.’s (2023) findings of sub-elite males who were not overrepresented. We did not find elite left-handed females to be overrepresented, which was at odds with Laxdal et al. (2023). Nevertheless, our findings were generally in line with those of previous investigators of left-handedness in various sports, where handedness was found to be less consequential for females than for males (e.g., Baker et al., 2013; Li, 2014; Loffing, Hagemann, & Strauss, 2012; Raymond et al., 1996; Richardson & Gilman, 2019). Packheiser et al. (2019) suggested that this may be because left-handed males are less lateralized than left-handed females.

Regarding the relationship between throwing arm and success from the 7-meter line, we found no indications of lateral biases. This failure to find a laterality performance bias for 7-meter throws is congruent with previous investigators’ results at various proficiency levels (Laxdal et al., 2022b, 2023) using different methodologies. Collectively, these results indicate that the advantages left-handers were presumed to have in the selection process do not translate to performance execution (i.e., success from 7-meter throws). In other words, the best right- and left-handed 7-meter shooters were similarly effective from the 7-meter line.

The selection of 7-meter shooters is somewhat opaque since most of the selection process takes place in training. Of course, players’ in-game performances are likely to impact their ranking (Laxdal et al., 2024), but the small number of eligible 7-meter shooters are usually predetermined by the coach. Eligible shooters have likely been taking 7-meter throws for most of their career, and their eligibility as 7-meter shooters in the games studied here was therefore, largely the result of previous selections that occurred earlier in their careers. Since left-handers have been found to have a greater advantage at the amateur level than at the professional level in tennis (Loffing, Hagemann, & Strauss, 2012), one could argue that 7-meter shooters’ youth records may be more heavily colored by lateral biases than these professional level records. In fact, many of the goalkeepers these 7-meter shooters played against during their formative years were likely to have much less relative experience playing against left-handers than professional goalkeepers have. But this is perhaps where comparing a specific action in a complex team sport to an action in individual contests like tennis or mixed martial arts comes up short.

Since Loffing (2017) found a positive relationship between time pressure and lateral biases in interactive sports, one would expect to see this effect in the 7-meter throw. The ball travels too fast for the goalkeeper to react to it, meaning that the goalkeeper must anticipate the ball’s trajectory, based on the shooter’s movements (Cocić et al., 2021; Schorer et al., 2018). In fact, Loffing et al. (2015) found that goalkeepers performed worse when trying to anticipate the throws of left-handers than the throws of right-handers. Interestingly, it was not the goalkeeper’s gaze behavior or the reaction time that appeared to be the deciding factor, but rather the time it took to interpret or categorize the throw information (Loffing et al., 2015).

Mesagno et al. (2019) found indications that left-handers were better equipped to handle pressure and were less impacted by high-pressure moments than their right-handed counterparts. Since 7-meter throws are inherently stressful, as all eyes are on the shooter and the expectancy of scoring is high, left-handers would be expected to perform better if their innate ability to thrive under pressure made them more likely to score. Then again, Bühren and Gabriel (2023) found that 7-meter shooters generally thrived under pressure. Their findings were corroborated by Debanne et al. (2018) who found that 7-meter shooters were not impacted by the weight of the situation and were equally effective, irrespective of the criticality of the shot.

### Limitations and Directions for Further Research

Our results should be viewed with our study’s limitations in mind. While sample sizes in both studies were relatively large, more datapoints might impact the results (see Richardson & Gilman, 2019). Future investigators might attempt to pool together the shooting records of players from even more leagues, though such publicly available longitudinal data on 7-meter shooting is sparse. As we compared the 7-meter records of players over several years, we could not control for temporal covariates such as age and experience. Many shooters in both samples shot only a few 7-meter shots during the studied periods. While this is a naturally occurring phenomenon, it leads to a skewed sample. Since a high scoring percentage from very few 7-meter shots is substantially different from a high scoring percentage from many 7-meter shots (i.e., 1/1 and 0/1 are not comparable to 40/40 and 0/40, even though both sets of examples represent 100% and 0%), we needed to account for this skewed data. Our sensitivity analyses applied for this purpose revealed no practically meaningful differences between these different groups. As previously mentioned, some 7-meter shots from low-frequency shooters in some earlier championships were not included in Study II. However, since the number of goals from low frequency scorers must be low, excluding their data is unlikely to have influenced these results.

## Conclusion

Our findings underline the complexities associated with studying handedness biases in sports. While being left-handed was not directly advantageous for successful 7-meter throws, the overrepresentation of left-handers among 7-meter throwers indicates that lateral biases played some role in the coaches’ selection processes. It is unclear whether the sex differences found in this study were context-dependent or related to the level of competition (i.e., elite vs. sub-elite).

## Data Availability

Data is openly available at https://doi.org/10.6084/m9.figshare.21026770.v3 and https://doi.org/10.6084/m9.figshare.24118353.v1. All data is based on material that is openly available and in the public domain.
